# Proteomics computational analyses suggest that the bornavirus glycoprotein is a class III viral fusion protein (γ penetrene)

**DOI:** 10.1186/1743-422X-6-145

**Published:** 2009-09-18

**Authors:** Courtney E Garry, Robert F Garry

**Affiliations:** 1 Department of Microbiology and Immunology, Tulane University Heath Sciences Center, New Orleans, Louisiana 70112, USA

## Abstract

**Background:**

Borna disease virus (BDV) is the type member of the Bornaviridae, a family of viruses that induce often fatal neurological diseases in horses, sheep and other animals, and have been proposed to have roles in certain psychiatric diseases of humans. The BDV glycoprotein (G) is an extensively glycosylated protein that migrates with an apparent molecular mass of 84,000 to 94,000 kilodaltons (kDa). BDV G is post-translationally cleaved by the cellular subtilisin-like protease furin into two subunits, a 41 kDa amino terminal protein GP1 and a 43 kDa carboxyl terminal protein GP2.

**Results:**

Class III viral fusion proteins (VFP) encoded by members of the *Rhabdoviridae, Herpesviridae *and *Baculoviridae *have an internal fusion domain comprised of beta sheets, other beta sheet domains, an extended alpha helical domain, a membrane proximal stem domain and a carboxyl terminal anchor. Proteomics computational analyses suggest that the structural/functional motifs that characterize class III VFP are located collinearly in BDV G. Structural models were established for BDV G based on the post-fusion structure of a prototypic class III VFP, vesicular stomatitis virus glycoprotein (VSV G).

**Conclusion:**

These results suggest that G encoded by members of the Bornavirdae are class III VFPs (gamma-penetrenes).

## Introduction

Members of the Bornaviridae are enveloped with nonsegmented negative-stranded RNA genomes. The type member is Borna disease virus (BDV), the causative agent of Borna disease, an often fatal neurological disease occurring mainly in horses and sheep in endemic regions of central Europe. The natural host range, prevalence, and geographic distribution of BDV are broad [1]. For example, recent studies demonstrated the existence of an avian reservoir of diverse bornaviruses [2] and provided evidence that bornaviruses are the etiologic agent for proventricular dilatation disease, a neuroinflammatory disease of psittacine birds [3]. It is yet to be established whether bornaviruses causes any overt disease in humans. However, correlative evidence exists linking BDV infection with neuropsychiatric disorders, such as bipolar disorder [4,5].

BDV is noncytolytic and highly neurotropic [6]. Although viral RNA and proteins are readily detectable in BDV-infected cells, production of cell-free virions or cell-associated infectivity is absent or extremely low [7]. BDV propagates via cell-to-cell spread within the central nervous system and cultured cells in the absence of detectable assembled virions [8,9]. It is likely that BDV initially interacts with the plasma membrane, followed by endocytosis and penetration by membrane fusion; cell-to-cell spread of bornaviruses may involve distinct mechanisms [10]. The BDV genome contains at least six open reading frames (ORF), and the product of BDV ORF IV corresponds to the type I surface glycoproteins found in other viruses of the order *Mononegavirales*. The BDV glycoprotein (G, GP-C, gp84) migrates due to extensive glycosylation with an apparent molecular mass of 84,000 to 94,000 kilodaltons (kDa). BDV G is post-translationally cleaved by the cellular subtilisin-like protease furin [11,10]. The amino terminal product (GP1, Gn, gp41) has an apparent molecular mass of 41 kDa. GP1 was initially difficult to detect in infected cells or virions due to its high glycan content, which shields antigenic sites from antibodies. However, a peptide-generated antibody was used to demonstrate the presence of GP1 in virions and infected cells [12]. The carboxyl terminal product, molecular mass 43 kDa (GP2, Gc, gp43), is less heavily glycosylated and readily detected in infected cells and virions. The uncleaved product G accumulates in the endoplasmic reticulum and perinuclear region. G is not detected on the plasma membrane, whereas GP2, but not GP1, accumulates at the cell surface [10]. There are conflicting results as to the presence of uncleaved G in virions [13,9]. GP2 contains several hydrophobic sequences, and appears capable of mediating cell:cell fusion in the absence of other BDV surface proteins [10,14]. GP1 linked to the vesicular stomatitis virus glycoprotein transmembrane domain (VSV G TM) domain is sufficient, in the absence of GP2, for receptor recognition, cell fusion and entry [15].

The structures of the bornavirus glycoproteins have not been determined, and it is unknown whether or not these proteins belong to any of the three known classes of proteins that mediate membrane fusion and entry of enveloped viruses. Orthomyxoviruses (excepting members of the *Thogotovirus *genus, which are insect pathogens), retroviruses, paramyxoviruses, filoviruses, arenaviruses, and coronaviruses encode class I viral fusion proteins (VFP, aka class I or α-penetrenes). Class I VFP contain a "fusion peptide," a cluster of hydrophobic and aromatic amino acids located at or near the amino terminus that initially interacts with the target cell membrane (plasma membrane or vesicle membrane) [16-23]. Most class I VFP have an aromatic amino acid (aa) rich pre-membrane domain, while all have a carboxyl terminal anchor. The post-fusion forms of class I viral fusion proteins have an extended amino terminal helix (N-helix, HR1), and a carboxyl terminal helix (C-helix, HR2) or "leash domain" that mediate trimerization.

Members of the *Flavivirus *genus of the *Flaviviridae *and the *Alphavirus *genus of the *Togaviridae *encode class II viral fusion proteins (class II or β-penetrenes) possessing three domains (I-III) of mostly antiparallel β-sheets, a membrane proximal α-helical stem domain and a carboxyl terminal anchor [24-26]. The fusion loops of class II VFP are internal and located in domain II, the fusion domain. *Hepaciviruses *and *Pestiviruses*, the two other genuses in the *Flaviviridae*, appear to encode truncated class II VFP [27]. Proteomics computational analyses and other studies suggest that the carboxyl terminal glycoproteins (Gc) of bunyaviruses, tenuiviruses and *Caenorhabditis elegans *retroviruses, are class II VFP [28,29].

The third class of VFP (class III or γ-penetrenes) is represented by glycoprotein (G) of rhabdoviruses and glycoprotein B (gB) of herpesviruses. Class III VFP contain a fusion domain, which is similar structurally to the fusion domains of class II VFP, other β-sheet domains, an extended α-helical domain in the post-fusion form, a membrane proximal stem and a carboxyl terminal anchor [30-32]. The extended α-helices in the post-fusion forms of rhabdovirus G and herpesvirus gB are involved in trimerization, a similar function served by the long α-helices in the post-fusion structures of class I VFP. Our prior proteomics computational analyses suggested that the fusion proteins of group I nucleopolyhedroviruses (NPV) of the Baculoviridae and members of the Thogotovirus genus of the Orthomyxoviridae, which together form the GP64 superfamily, are also class III VFP [33]. A recent X-ray crystallographic study, published after our prior manuscript, confirmed that GP64 superfamily members are class III VFP [34]. Here, proteomics computational analyses are presented suggesting that bornavirus G are class III VFP.

## Materials and methods

### Sequences

Sequence and structural comparisons were performed for Borna disease virus strain V glycoprotein isolated from a horse (accession number NP042023). Additional bornavirus G sequences used in the analyses included ABH03174, CAC70658 (horse), AAL49985 (sheep), ABH03169 (rabbit), ACG59352 (avian - *Aratinga solstitialis*), and AAA91195 (human). We also made comparisons of bornavirus G with Thogato virus strain SiAr 126 envelope glycoprotein precursor (P28977), the *Autographa california *multiple nucleopolyhedrovirus GP64 superfamily protein (P17501) and other GP64 superfamily members. Representatives of G from six genera of the *Rhabdoviridae *were also used for sequence and structural comparisons: *Vesiculovirus*: VSV strain Indiana (AAA48370); *Lyssavirus*: rabiesvirus strain street (AAA47211); *Ephemerovirus*: bovine ephemeral fever virus structural G (P32595) and nonstructural G(P32596); *Novirhabdovirus*: infectious hematopoietic necrosis virus (CAA61498); *Cytorhabdovirus*: lettuce necrosis yellows virus glycoprotein (LYP425091); *Nucleorhabdovirus*: rice yellow stunt virus (AB011257) and an unclassified rhabdovirus: Taastrup virus (AY423355). We also compared bornavirus G to VFP of representative members of the *Herpesviridae*, *Flaviviridae, Togaviridae*, and *Bunyaviridae*.

### Proteomics computational methods

Methods developed by William Gallaher and coworkers to derive models of VFP have been described previously [22,18,17,20]. William Pearson's LALIGN program , which implements a linear-space local similarity algorithm [35] was used to perform regional alignments. PHD (Columbia University Bioinformatics Center, ), which is part of the ProteinPredict suite was the preferred method of secondary structure prediction [36]. Domains with significant propensity to form transmembrane helices were identified with TMpred (ExPASy, Swiss Institute of Bioinformatics, ). TMpred is based on a statistical analysis of TMbase, a database of naturally occurring transmembrane glycoproteins [37]. Sequences with propensity to interface with a lipid bilayer were identified with Membrane Protein e**X**plorer version 3.0 from the Stephen White laboratory using default settings [38], which can be used to calculate scores on the Wimley-White interfacial hydrophobicity scale (WWIHS) [39]. Figures were drawn using Freehand (Macromedia).

## Results

### Similar sequences and common structural/functional motifs are located collinearly in VSV and BDV G

**Figure 1:**
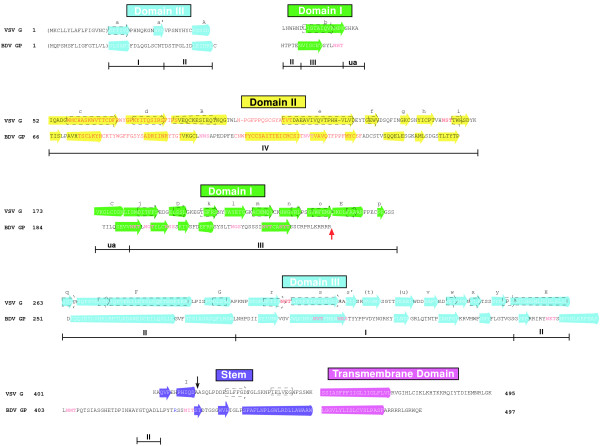
**Collinear arrangement of similar structures in BDV G and VSV G**. The post fusion secondary structure of VSV G as solved and numbered by Roche and coworkers [30] is depicted with α-helices as cylinders and β-sheets as arrows. The α-helices predicted by PHD In BDV G are indicated similarly. β-sheets (t) and (u) of VSV G are not present in the protein data base structure (2cmz.pdb). In VSV G, α-helices predicted by PHD are indicated by dashed boxes and predicted β-sheets are identified with dashed arrows. Amino acids are numbered beginning after the putative signal sequences enclosed in parentheses. Plum amino acids: N-glycosylation sites. Sequences with significant WWIHS scores in the fusion domain (II) were identified by MPeX and colored red. Hydrophobic transmembrane domains (violet) were predicted using TMpred. A class III domain nomenclature is used here that can apply to both class II and III VFP: domain I (green), domain II (yellow), domain III (blue), and stem domain (indigo). This unified nomenclature assigns domain II (IV in the VSV G nomenclature of Roche et al. [30], I in the HSV-1 gB nomenclature of Heldwein et al. [32] and Ia and Ib in the baculovirus nomenclature of Kadlec et al. [34]) as the class III fusion domain as in class II VFPs. In addition to minor adjustments in the ends of domains, the current class III VFP numbering also combines two interacting domains into domain III (I + II in Roche's VSV G nomenclature, III + IV in Heldwein's HSV-1 gB nomenclature and Kadlec's baculovirus nomenclature). The domain numbering originally proposed is also indicated. UA represents "hinge" aa not assigned to domains in VSV G in the prior scheme.

Proteomics computational tools have been applied successfully to discover potential structures of the VFP of retroviruses, filoviruses, coronaviruses, and baculoviruses [22,17,18,20,21,27,28,33]. Subsequent studies, using X-ray crystallography and other methods, confirmed all essential features of our structural models suggesting that these modeling methods are highly robust [19,40,34]. The PHD algorithm predicts protein secondary structure from multiple sequence alignments by a system of neural networks, and is rated at an expected average accuracy of 72% for three states, helix, strand and loop [36]. This algorithm predicts that there is a region that forms an extended α-helix in BVD G (aa 284-338) and other bornaviruses (not shown). A prominent feature of class III VFPs is an extended α-helix beginning near the carboxyl terminal third of the ectodomain (helix F in domain III, Fig 1), which is involved in trimerization of the post-fusion structure [30,32,34]. The longest α-helix predicted by PHD in BVD G corresponds to this location (Fig. 1, aa 251-280). Two shorter helices corresponding to helices G and H of VSV G are also predicted by the PHD algorithm in BVD G (aa 284-297, 392-402). With the exception of these α-helices, the ectodomains of BVD G are predicted to be comprised mostly of β-sheets, as is the case with other class III VFP.

Another domain identifiable with computational tools in BVD G is the carboxyl terminal transmembrane anchor. TMpred, an algorithm that identifies possible transmembrane helices, assigns a significant score (2578, >500 is statistically significant) to BVD G aa 468-493, which suggests that this sequence includes the transmembrane anchor (violet cones). Two other potential TM segments are present in BDV G (274-294 and 303-327). However, it is not likely that these regions are embedded in the membrane, either in G or GP2 because of the likely dicysteine bonding between cysteines 272 and 317 or other potential linkages involving cysteine 317. PHD analyses also predict the presence of an α-helical stem domain with several aromatic aa prior to the transmembrane anchor (aa 445-474), a feature present in both class II and III VFP [41,42]. Therefore, we have designated the sequence 475-493 as the transmembrane anchor, recognizing that the stem domain, which contains several aromatic amino acids, is likely to interface with the viral membrane.

The fusion domains (referred to here as domain II) of all class II or III VFP contain 1 or 2 fusion loops, which give significant scores on the WWIHS [39]. Sequences with positive WWIHS have a high potential to interface with or disrupt lipid membranes, and therefore are key features of VFP. Another feature of the fusion domains of class II and III VFP is the presence of several dicysteine bonds, which stabilize the overall domain architecture. A region in bornavirus G (BVD aa 66-183) with 12 cysteine residues, plus 2 sequences with positive WWIHS scores (Fig. 1, red, 74-102, 119-155) is likely to represent the fusion domain of BDV G.

**Figure 2:**
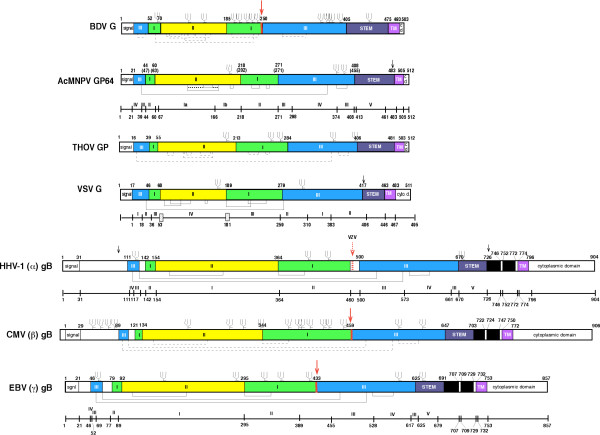
**Similar linear arrangement of putative domain structures of BDV G, VSV G, THOV GP and AcMNPV GP64 and herpesvirus gB**. Class III VFP domain nomenclature and coloring is as in Fig. 1. Amino acids are numbered beginning after the putative signal sequences in VSV G, but at the beginning of the signal sequence of HSV-1 gB. Arrows indicate G and gB truncations of the forms used for crystallography. Solid lines represent cysteine bonding in VSV G, AcMNPV GP64, and HSV-1 and EBV gB [30,32,34,43]. Black boxes represent hydrophobic regions, with violet representing the transmembrane anchor (TM). Dashed lines represent potential cysteine bonding in BDV G, THOV GP and CMV gB. Dashed arrow is the location of a furin cleavage site in the alphaherpesvirus varicella zoster virus (human herpes virus 3). Solid arrows are furin cleavage sites in BDV G, CMV gB, and EBV gB.

Sequence similarities between VSV and BDV G do not permit alignment by computational methods alone. However, using the regions of local structural similarity including the putative fusion domain/loops, extended α-helices and transmembrane domains, all of which are collinear, alignments between VSV and BDV G are proposed (Figs. 1, 2). These alignments support assignment of a class III domain architecture to BDV G. The proposed domains of bornavirus G are also collinear with analogous domains of herpesvirus gB, the other - albeit much longer - prototypic class III VFP, as well as GP64 superfamily proteins encoded by baculoviruses and thogotoviruses (Fig. 2).

BDV G is more heavily glycosylated than VSV G, THOV G or baculovirus GP64, It is also more heavily glycosylated than gB of HSV-1 (Fig. 2). Cytomegalovirus (CMV) and Epstein-Barr virus (EBV) gB are closely related in sequence to HSV-1 gB. Recent studies confirm that EBV gB is a class II VFP [43], and it is likely that all herpesvirus gB are class II VFP. CMV gB is glycosylated to a similar extent as BDV G, while EBV gB has an intermediate level of glycosylation. Therefore, a high level of glycosylation does not preclude the possibility that BDV G is a class III VFP.

BDV G and other bornavirus G have consensus furin cleavage sites (RRRR) prior to the extended α helix (Fig. 1, 2) that are utilized in infected cells to cleave the proteins into two subunits GP1 and GP2 [11,9]. Several alphaherpesviruses, such as varicella zoster virus (VZV), posses a furin cleavage site in the analogous location (Fig. 2 dashed orange arrow). Most beta and gamma-herpesviruses, including CMV and EBV, have a furin cleavage site prior to the extended α-helix. Therefore, possession of a furin cleavage site does not preclude the possibility that BDV GP is a class III VFP.

**Figure 3:**
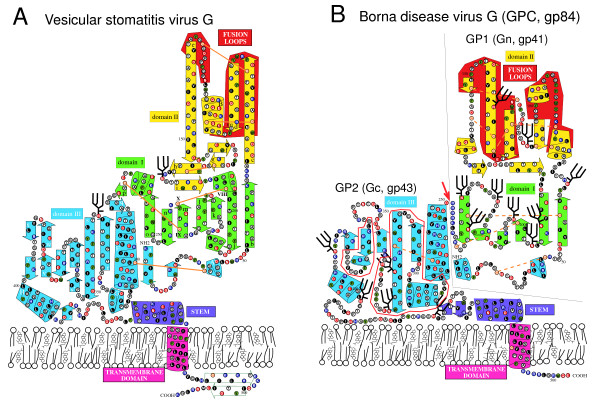
**Model of BDV G based on the X-ray crystallographic structure of VSV G**. The predicted structures of BDV G was fit to the post-fusion structure of VSV G [30]. The post fusion secondary structure of VSV G as solved by Roche and coworkers [30] is depicted with α-helices as cylinders and β-sheets as arrows. β-sheets (t) and (u) of VSV G are not present in the protein data base structure (2cmz.pdb). Sequences with significant WWIHS scores were identified by MPeX and filled red or outlined with red lines. Hydrophobic transmembrane domains (violet) were predicted using TMpred as discussed in the text. Secondary structures for BDV G were predicted by PHD. Orange/black lines: dicysteine linkages. Black stick figures: N-glycosylation sites.

Cysteine residues are usually the most conserved aa within a protein family because disulfide bonds between cysteines are critical determinants of secondary structure. The cysteines of class III (and class II) VFPs determined by X-ray crystallography are arranged such that disulfide bonds are formed between cysteine residues within the same domain. To determine the plausibility of the proposed alignment, a model of BDV G scaffolded on the structure of VSV G in the post-fusion (low pH) configuration [30] was constructed (Fig. 3). The alignment between VSV and BDV G suggest that these VFPs may have a similar structure. Therefore, putative structures in BDV G are depicted as in VSV G. The proposed BDV G model is based principally on the structural predictions of PHD, the most robust secondary structure prediction algorithm used. These results provide evidence that the 10 cysteines in the putative fusion domain (domain II) can potentially bond. Such linkages can stabilize the fusion loops as occurs in both class II and III VFP. The dicysteine linkages are modeled such that all cysteine bonding occurs between the putative domains, as is the case with other class III VFP. After cleavage by furin GP1 and GP2 of BDV appear not to be linked by covalent bonding, as the subunits disassociate during SDS-PAGE [9]. Therefore, we have not modeled any dicysteine linkages between the GP1 and GP2 subunits. There are plausible intradomain linkages that can form between each of the cysteines in BDV G.

The BDV G structural model presented here is not intended as a definitive structural prediction. Rather, there are many possible alternatives to the secondary and tertiary structures and the cysteine linkages of BDV G. For example, it is possible that in a minor subset of G may be cross-linked via cysteine binding such that the GP1 and 2 subunits are covalently bound. The modeling does establish that feasible structures exist that are consistent with the secondary structure predictions and with the assignment of BDV G as a class III VFP.

## Discussion

Proteomics computational analyses suggest that bornavirus G are class III VFP. Computational analyses and other methods predict that each of the major features common to class III fusion proteins are present in BVD G, including internal fusion loops, an extended α helical domain, a stem domain and a carboxyl terminal transmembrane domain. These features of BDV G are located collinearly with those of VSV G, a prototypic class III VFP [30,31]. On the basis of sequence similarities amongst G of members of the *Bornaviridae *it is likely that all are class III VFP. Structural models including feasible cysteine linkage maps could be readily established for BDV G using the VSV G post-fusion structure as a scaffold. The fusion domains of BVD G, which we refer to here in a unified domain nomenclature as domain II, appear to be stabilized by cysteine bonds and to contain one or more loops with positive WWIHS scores, features characteristic of the fusion domains of both class II and III VFP. Differences between VSV and BDV G include the presence of a consensus furin cleavage site and a higher number of N-glycosylation sites in BDV G. However, many herpesvirus gB, which are class III VFP, contain a furin cleavage domain prior to the extended α helix in domain III and are more heavily glycosylated than VSV G. Therefore, the presence of the furin cleavage site and high glycan content in BDV G does not preclude its putative inclusion in class III. Whether or not the secondary and tertiary folding of BDV GP conforms to the domain structure of class III VFP will require x-ray crystallographic or other physical structural determinations.

The three VFP classes for enveloped virus membrane glycoproteins were established based on structural similarities in the post-fusion configurations. Therefore, it is likely that there is a common post-fusion (low pH) configuration of class III VFP, and that BDV G has a post-fusion structure similar to VSV G. In contrast, the prefusion configurations of class I, II and II VFPs are highly variable. The virion configuration of VSV G is homotrimer arranged in a tripod shape with the fusion domains corresponding to the legs of the tripod [31]. No structural prediction of the prefusion configurations of BVD GP is possible.

As in the case of class I fusion proteins, BDV G is cleaved by a cellular protease into two subunits [11,9]. It has been suggested that hydrophobic sequences following this cleavage site near the GP2 N terminus may function as a fusion peptide [11]. The current modeling suggests that bornavirus G is not a class I VFP, and does not corroborate the existence of a "fusion peptide" in GP2. The position of predicted α helices, dicysteine linkages and membrane interactive regions (sequences with positive WWIHS scores) are not consistent with assignment of BDV G to class I. However, the concept that multiple domains of bornavirus G participate in fusion of viral and cellular membranes is consistent with current viral entry models. In class I VFP both the amino terminal fusion peptide, the pre-membrane aromatic domain and the TM cooperate to mediate lipid mixing. Likewise, in class II and III VFP, fusion loops in domain II, the stem domain, TM and other WWIHS score positive sequences all appear to participate in fusion. Several of the domains of GP1 and GP2, particularly the putative fusion loops (domain II), the sequences with positive WWIHS scores in GP2, and the stem and TM domain could cooperate or interact to mediate fusion and entry of BDV.

BDV entry is via clathrin-mediated endocytosis, and the fusion between viral and cellular membranes occurs in the mildly acidic environment of the early endosome [44]. The domains of BDV G involved in receptor recognition and cell entry have not been defined. GP1 sequences are capable of mediating attachment and entry in VSV pseudoparticles in the absence of GP2 [15]. BDV-infected cells exhibit syncytium formation upon exposure to low-pH medium [10]. This pH-dependent cell fusion event is likely mediated by GP2 since it is the only membrane-anchored BDV glycoprotein found on the plasma membrane. These results suggest that both GP1 and GP2 are involved in membrane fusion, either cooperatively or in the case of cell surface expressed GP2 independently. An analogous situation may exist for hepatitis C virus (HCV). Our previous modeling suggested that the envelope proteins of HCV split the duties performed by envelope proteins of other members of the Flaviviridae, E of flaviviruses and E2 of pestiviruses [27]. HCV E1 contains the fusion loops that are analogous to the domain II fusion loops of E/E2, while HCV E2 contains receptor binding domains analogous to domain III of E/E2, as well as the stem domain.

The BDV cellular receptor(s) has not been identified. Baculoviruses, which have a broad host range, may not possess a specific protein receptor. Rather, it has been suggested that the fusion loops (domain II) of baculovirus gp64, a class III VFP, may be the initial binding point with lipids of the target cell membrane [34]. The host range of bornaviruses also appears to be broad. Whether bornavirus G utilizes a specific protein receptor or bind to lipids or other ubiquitous components of cellular membranes remains to be determined.

*Orthomyxoviridae*, *Retroviridae*, *Paramyxoviridae*, *Filoviridae*, *Arenaviridae*, and *Coronaviridae *and *Baculoviridae *have members that encode class I VFP [16,22-21,45]. *Flaviviridae*, *Togaviridae*, and *Bunyaviridae *family members are known or appear to have members that encode class II VFP [24,27-29]. If the current analyses are correct, BDV G joins rhabdovirus G, herpeviruses gB, thogotovirus G and baculovirus GP64 and as a class III VFP. While convergence to common structures is possible, VFP of enveloped viruses may have evolved from a limited number of common progenitors. Support for this hypothesis comes from the remarkable similarities in the post-fusion structures of the VFP in each class, even though the proteins differ dramatically in aa sequence. While, it is probable that other classes of VFP exist, there appears be a limited number of effective structures for virus-mediated membrane fusion.

**Figure 4:**
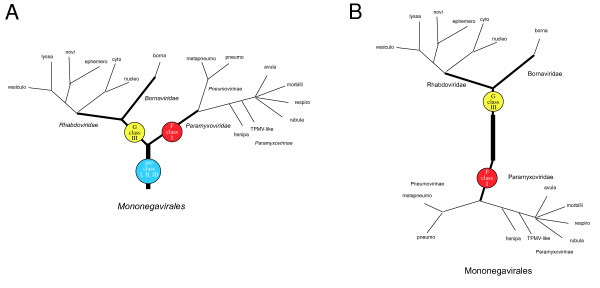
**Acquisition of class I or III VFP by members of the *order Mononegavirales***. Thick lines indicate primordial lineages and thin lines are lineages leading to contemporary viruses. The alternative in Panel A shows a tree that is rooted, with the glycoproteins of all viral families evolved from a common class I, II and III progenitor. Panel B shows another possible evolutionary tree, which is unrooted, and depicts independent acquisitions of class I VFP by an ancestral paramyxoviruses and class III VFP of a common ancestor of rhabdoviruses and bornaviruses. A variation of this former tree (not shown) would involve separate acquisitions of class III VFP by ancestral rhabdoviruses and bornaviruses.

The Bornaviridae are included with the Rhabdovirdae and Paramyxoviridae in the order Mononegavirales. Paramyxoviruses possess a class I VFP, whereas rhabdoviruses and, as suggested here, bornaviruses encode class III VFP. There are several possibilities for how the glycoproteins of members of this order evolved. A G gene appears to have been present in the common ancestors of all members of the *Rhabdoviridae and Bornaviridae*. The similarities detected between rhabdovirus and bornavirus G are consistent with divergent evolution from a common progenitor, but sequence similarities are insufficient to establish a phylogenic relationship. Therefore, it is possible that the class III VFP of rhabdoviruses and bornaviruses were acquired by independent genetic events. An alternative suggested by Kadlec et al. [34] is that the three classes of VFP may have evolved from a common precursor (pre-class I, II, III). This concept is based on morph videos and other analyses that reveal domain-specific folding and structural similarities amongst each of the three classes of VFP. If this is the case, mononegavirales evolution can be depicted as a "rooted" tree with the ancestral mononegavirus possessing a progenitor of class I, III and likely II VFP (Fig. 4A). Alternatively, the VFP of paramyxoviruses could have been acquired independently from the acquisition of glycoproteins by rhabdoviruses and bornaviruses by horizontal gene transfer. This case can be depicted as an unrooted tree, in which glycoproteins were acquired after divergent of paramyxoviruses from rhabdovirus and bornaviruses (Fig. 4B). VFP are highly divergent at the primary sequence level. Therefore, definitive statistical analyses of these possibilities are not possible at this time. The evolutionary origins of VFP, which display many common structural features, offer worthy challenges to computational biologists.

In the absence of determinations by X-ray crystallography, structural models such as the one proposed here can provide useful hypotheses to guide experimental strategies for development of vaccines or drugs to prevent or treat infection by bornavirus infections. Prior to the availability of X-ray structural data, several potent HIV-1 entry inhibitors were developed [46,47] based on the Gallaher HIV-1 TM fusion protein model [17]. Fuzeon™ (DP178; T20 enfuvirtide), one of these peptides corresponding to a portion of the carboxyl terminal helix and the pre-anchor domain in this class I VFP, has been shown to substantially reduce HIV-1 load in AIDS patients, and is well-established in the treatment of HIV infection in the United States and European Union [48]. Peptide entry inhibitors of viruses with class II VFP have also been developed [49], and have also been described for the class III VFP of herpesviruses (Melnik et al., in preparation). Given that bornaviruses cause a range of infectious neurological syndromes in warm-blooded animals, with high mortality rates, veterinary applications of bornavirus entry inhibitors, should be investigated. Confirmation of the still controversial proposal that bornaviruses cause neuropsyciatric disorders in humans would provide additional strong incentives to develop preventative vaccines or therapeutics.

## Competing interests

The authors declare that they have no competing interests.

## Authors' contributions

CEG performed sequence alignments, and assisted in preparation of figures. RFG wrote the manuscript. Both authors read and approved the final manuscript.
